# Contact dermatitis presenting as non-healing wound: case report

**DOI:** 10.1186/1447-056X-10-6

**Published:** 2011-05-15

**Authors:** M Leelavathi, YY Le, H Tohid, AH Hasliza

**Affiliations:** 1 Department of Family Medicine, Faculty of Medicine, Universiti Kebangsaan Malaysia; 2 Dermatology Unit, Department of Medicine, Faculty of Medicine, Universiti Malaya

## Abstract

Topical antiseptics are commonly used in the management of minor wounds, burns, and infected skin. These agents are widely used by health professionals and are often self-prescribed by patients as they are easily available over-the-counter. This case illustrates a 73 year old man who presented with a non-healing wound on his right forearm for 4 weeks. The wound started from an insect bite and progressively enlarged with increasing pruritus and burning sensation. Clinically an ill-defined ulcer with surrounding erythema and erosion was noted. There was a yellow crust overlying the center of the ulcer and the periphery was scaly. Further inquiry revealed history of self treatment with a yellow solution to clean his wound for 3 weeks. Patient was provisionally diagnosed to have allergic contact dermatitis secondary to acriflavine. Topical acriflavine was stopped and the ulcer resolved after treatment with non-occlusive saline dressing. Skin patch test which is the gold standard for detection and confirmation of contact dermatitis showed a positive reaction (2+) to acriflavine. Acriflavine is widely used as a topical antiseptic agent in this part of the world. Hence, primary care physicians managing a large variety of poorly healing wounds should consider the possibility of contact allergy in recalcitrant cases, not responding to conventional treatment. Patient education is an important aspect of management as this would help curb the incidence of future contact allergies.

## Background

Acriflavine lotion is a topical antiseptic solution yellow or orange in colour, mainly used for minor wounds, burns, and infected skin. Although used in dilution (0.1%) for medical purposes, this agent has been documented to produce potential skin itchiness, irritation or burning sensation upon contact. Nevertheless, it is still widely used for wound dressing by health professionals and remains a popular topical antiseptic agent purchased over-the-counter.

## Case presentation

**Figure 1:**
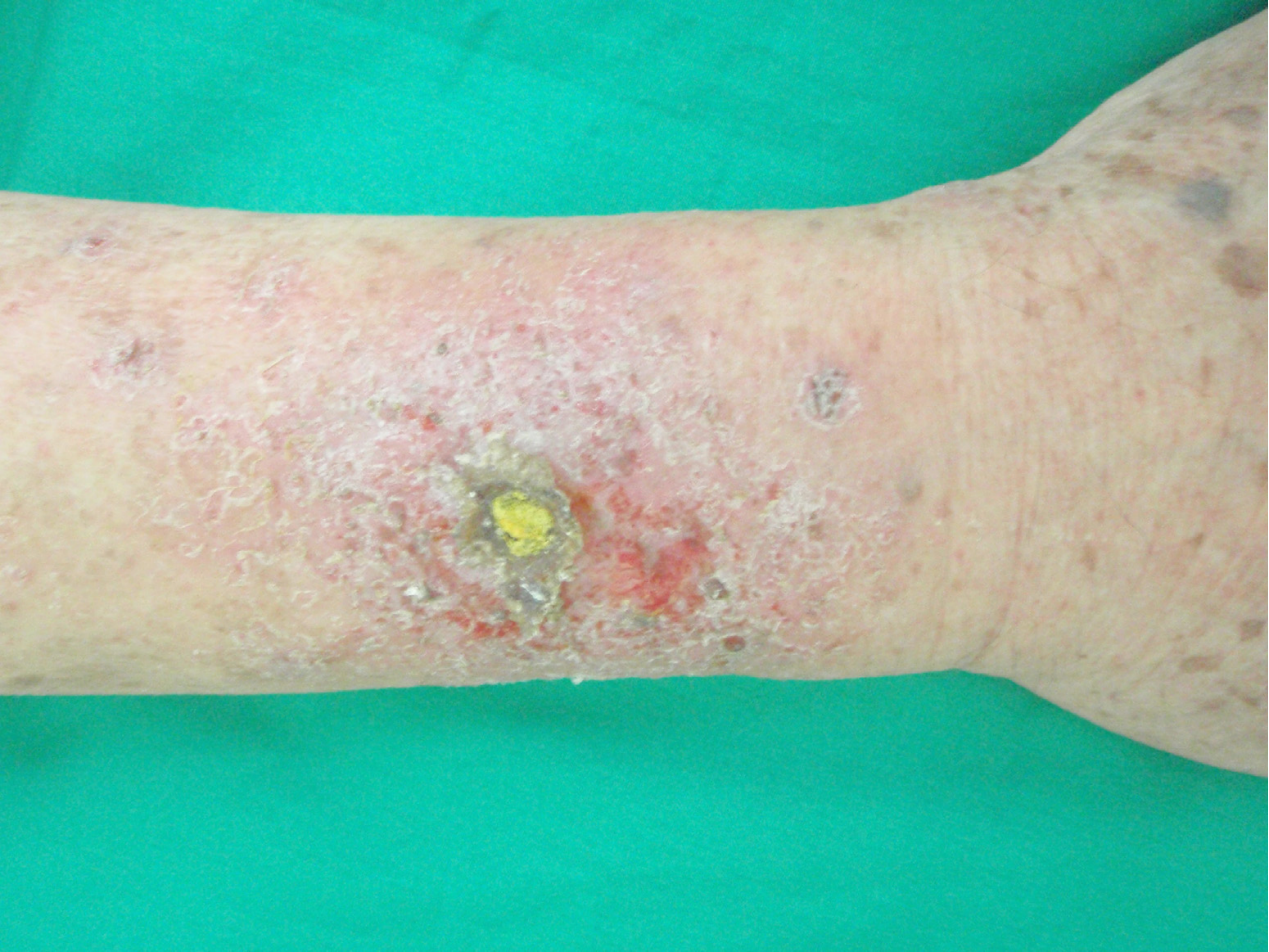
**Right forearm showing ulcer with central yellow crust surrounded by erythema and erosion**.

**Figure 2:**
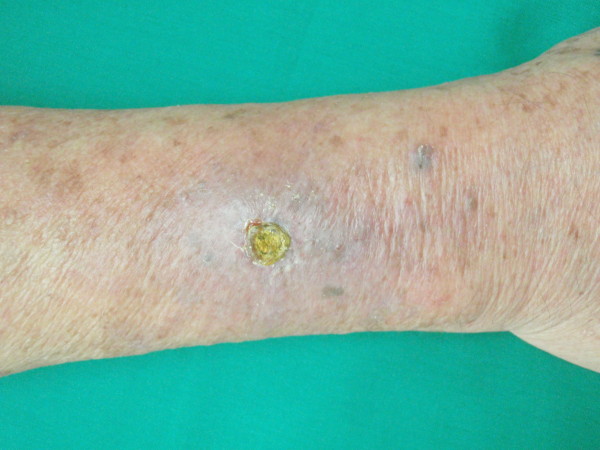
**The ulcer is dry, less inflamed and smaller a week after withholding acriflavin**.

We report a case of a 73 year old man presenting with a non healing wound on his right forearm for 4 weeks. The wound started from an insect bite and progressively enlarged with increasing pruritus and burning sensation. Clinically an ill-defined ulcer was seen on his right forearm with surrounding erythema and erosion. There was a yellow crust overlying the center of the ulcer and the periphery was scaly (Figure 1). Further inquiry revealed history of self treatment with a yellow solution to clean his wound for 3 weeks. He could not recall using the solution in the past and did not seek any medical advice prior to the current visit. Patient was provisionally diagnosed to have allergic contact dermatitis secondary to acriflavine. Acriflavine usage was stopped and the ulcer was treated with daily non-occlusive saline dressing. The lesion improved a week later looking noticeably dry, less inflamed and smaller in diameter (Figure 2). At the end of two weeks, there was complete resolution of the ulcer, leaving a residual post-inflammatory hyperpigmentation. A patch test using Standard European series (*Chemotechnique*) containing 28 different allergens and acriflavine was done seven months later. The result showed a positive reaction (2+) to acriflavine while the rest were negative.

Acriflavine or commonly known as Flavin, is an acridine derivative of proflavin used as a topical antiseptic agent. It is a yellow or orange colour solution which stains the skin and may cause irritation, inflammation or blister upon contact [1]. It is commonly used as skin disinfectant for minor wounds, burns, infected skin and is effective against both gram positive and negative bacteria [1]. Acriflavine was initially used during the First World War as a treatment for African trypanosomiasis (sleeping sickness). In addition to its disinfecting property, it has also been shown to inhibit cancer progression in animals [2]. Although used in dilution (0.1%), it has been documented to produce potential skin irritation and is still widely used for wound dressing in both hospitals and outpatient clinics.

Contact dermatitis is an eczematous symptom occurring as a result of skin exposure to an irritant or sensitizing agent. It is generally categorized as allergic contact dermatitis and irritant contact dermatitis. Allergic contact dermatitis is an immune mediated inflammatory reaction while irritant contact dermatitis is a non-allergic inflammatory reaction causing direct cell damage resulting in skin dryness, redness or even burns [3]. Common antibacterials and antiseptics that cause an allergic reaction are Neomycin (40.6%), Soframycin (15.1%), Dettol (10.9%), Savlon (8.3%) and Acriflavine (5.2%) [4,5]. Besides contact dermatitis, acriflavine has also been shown to cause perioral and mucosal odema [6].

Individuals of all age groups and ethnicity have potential risk for developing contact dermatitis ranging from diaper dermatitis in infants to hair dye and fragrance dermatitis in the elderly [1]. Various studies have shown increasing prevalence of contact dermatitis to topical medicaments with advancing age [7,8]. This is probably due to the fact the elderly have been exposed to multiple allergens during their life time compared to younger individuals. Atopic individuals were previously thought to be more susceptible to contact dermatitis. However, a definite relationship between atopic dermatitis and contact dermatitis is yet to be established as current evidence show controversial results [1].

Typical features of dermatitits medicamentosa are non healing wound, worsening or enlarging wound with presence of surrounding dermatitis. Staining of the wound provides a clue to identify the irritant; yellow (acriflavine) or dark brown (potassium permanganate). The reaction is usually gradual and not an acute event. Hence patients are usually unaware of the underlying cause for the reaction which further delays wound healing. Withholding the irritant and appropriate topical management speeds up recovery.

Patch test is the gold standard for detection and confirmation of contact dermatitis. This is a simple yet cost effective test that facilitates early and accurate detection of underlying cause for suspected contact dermatitis [9]. It also serves as an important tool in patient education to avoid further exposure to the known allergens. Although the test is simple to perform, adequate experience and training is required for accurate interpretation of the results. Patch test is best avoided during an acute reaction and performed about six months after an initial contact to prevent further aggravation of the existing dermatitis [1].

## Conclusion

Acriflavine remains as a widely used topical antiseptic agent in this part of the world. Hence, primary care physicians managing a large variety of poorly healing wounds should consider the possibility of contact allergy in recalcitrant cases, not responding to conventional treatment. A detailed history of the wound, from the initial onset till presentation and the different types of treatments, either from their own kitchen (home remedies), over-the-counter purchase and type of dressing solutions used should be inquired. History of previous local reactions to antiseptic solutions and possible cross reactions should also be clearly documented. Patient education is an important aspect of management as this would help curb incidence of future contact allergies.

## Consent statement

Written informed consent was obtained from the patient for publication of this case report and accompanying images. A copy of the written consent is available for review by the Editor-in-Chief of this journal.

## Competing interests

The authors declare that they have no competing interests.

## Authors' contributions

LM contributed in management of the patient till recovery and has the main role in producing this manuscript

LYY contributed in interpretation of the patch test, literature review and drafting of this manuscript

TH participated in patient management and drafting of this manuscript

HAH participated in coordination of patient care and drafting of this manuscript

All authors have read and approved the final manuscript.

## Authors' information

The author is a registered Family Medicine Specialist who has special interest in the field of Dermatology. The author has successfully completed the Graduate Diploma in Family Practice Dermatology from the National Skin Centre in Singapore and spent one year full time attachment at the Dermatology Department of Kuala Lumpur General Hospital, Malaysia.
